# Altered Processing and Integration of Multisensory Bodily Representations and Signals in Eating Disorders: A Possible Path Toward the Understanding of Their Underlying Causes

**DOI:** 10.3389/fnhum.2018.00049

**Published:** 2018-02-12

**Authors:** Giuseppe Riva, Antonios Dakanalis

**Affiliations:** ^1^Centro Studi e Ricerche di Psicologia della Comunicazione, Università Cattolica del Sacro Cuore, Milan, Italy; ^2^Applied Technology for Neuro-Psychology Laboratory, Istituto Auxologico Italiano (IRCCS), Milan, Italy; ^3^Department of Medicine and Surgery, Università degli Studi di Milano Bicocca, Milan, Italy; ^4^Department of Brain and Behavioral Sciences, University of Pavia, Pavia, Italy

**Keywords:** multisensory body integration, eating disorders, self-objectification, interoception, body dissatisfaction, virtual reality, body memory, body representations

## Abstract

According to the Diagnostic and Statistical Manual of Mental Disorders (DSM V) eating problems are the clinical core of eating disorders (EDs). However, the importance of shape and weight overvaluation symptoms in these disorders underlines the critical role of the experience of the body in the etiology of EDs. This article suggests that the transdiagnostic centrality of these symptoms in individuals with EDs may reflect a deficit in the processing and integration of multisensory bodily representations and signals. Multisensory body integration is a critical cognitive and perceptual process, allowing the individual to protect and extend her/his boundaries at both the homeostatic and psychological levels. To achieve this goal the brain integrates sensory data arriving from real-time multiple sensory modalities and internal bodily information with predictions made using the stored information about the body from conceptual, perceptual, and episodic memory. In this view the emotional, visual, tactile, proprioceptive and interoceptive deficits reported by many authors in individuals with EDs may reflect a broader impairment in multisensory body integration that affects the individual’s abilities: (a) to identify the relevant interoceptive signals that predict potential pleasant (or aversive) consequences; and (b) to modify/correct the autobiographical allocentric (observer view) memories of body related events (self-objectified memories). Based on this view, the article also proposes a strategy, based on new technologies (i.e., virtual reality and brain/body stimulation), for using crossmodal associations to reactivate and correct the multisensory body integration processes.

## Introduction

The American Psychiatric Association’s (APA) Diagnostic and Statistical Manual of Mental Disorders (DSM V) identifies eating problems as the clinical core of eating disorders (EDs). However, the importance of shape and weight overvaluation symptoms in these disorders underlines the critical role of the experience of the body in their etiology. This vision is strengthened by the results of two 4-year longitudinal studies—the first involving 2713 female college students (Dakanalis et al., 2017a), and the second including 2507 male college students (Dakanalis et al., 2016c)—recently completed by our team. Both studies explored the role played by theoretically relevant factors in predicting the onset and maintenance of EDs. In both samples self-objectification, body dissatisfaction, appearance-ideal internalization, dieting and negative affectivity at baseline, as well as changes in these factors, predicted onset and maintenance of DSM-5 EDs at 4-year follow-up. Even if all these vulnerability factors are traditionally accepted as having a critical role in the emergence and maintenance of EDs (Calogero et al., 2010; Culbert et al., 2015) the newness of the two studies is the different predictive value of the assessed factors. First, the relative variance explained by body dissatisfaction and appearance-ideal internalization (the extent to which one sets culturally defined appearance ideals as his/her own personal standard of attractiveness) is almost two times the variance explained by dieting and by negative affectivity. Furthermore, the relative variance explained by self-objectification (the tendency to experience one’s body from an external observer’s perspective) is almost four times the variance explained by dieting, and more than four time the variance explained by negative affectivity, at least for females.

Apparently, these and other results (Dakanalis et al., 2014, 2015a,c, 2016a,d; Kearney-Cooke and Tieger, 2015) suggests an important role of the experience of the body in the emergence and maintenance of EDs (Dakanalis et al., 2016b), even if the causal path has not been fully elucidated, in particular for binge-eating subjects (Grilo et al., 2005; Herbozo et al., 2015).

More, these results are in line with the tenets of the Objectification Theory (Fredrickson and Roberts, 1997). This theory suggests that the pressure to constantly monitor and scrutinize our body from outside, to ensure its conformity with cultural standards (Calogero et al., 2017), encourages specific cognitions and behaviors that leads to increased body shame and subsequent EDs (Fitzsimmons-Craft et al., 2011; Kroon Van Diest and Perez, 2013; Dakanalis et al., 2017c). However, the situation is more complex than it might seem to be at first sight (Dakanalis et al., 2015b). As recently reported by Holland et al. (2017), objectifying events are frequently experienced by young women: the authors reported that women experienced objectifying events approximately once every 2 days and witnessed them more than once per day. The pervasivity of this experience is also reflected by the level of dissatisfaction: two online cross-sectional studies (Runfola et al., 2013), the UNC-SELF study and the Gender and Body Image Study (GABI), involving more than 5868 women residing in the United States, found that 90.0% of the women in the 25–34 age bracket and 93.2% in the 35–44 age bracket experienced body dissatisfaction. Nevertheless, the percentage of female individuals who met DSM-5 criteria for an ED diagnosis is much lower: in our samples 13.1% of college women at baseline and 7.6% at 4-year follow-up.

Even if self-objectification is a critical risk factor for the development of EDs, the Objectification Theory is still not able to answer two critical questions: Why do not all the individuals experiencing self-objectification develop EDs? What is the role of the body experience in the etiology of these disorders?

Here we will embrace an emerging field of neuroscience—the multisensory integration of bodily representations and signals—to answer the above questions. Specifically, the first goal of this manuscript is to describe a plausible, testable and transdiagnostic model of EDs centered on multisensory body integration deficits. Secondly, we also suggest possible techniques based on the presented model to correct these deficits.

## Multisensory Body Integration in Brief

**Figure 1:**
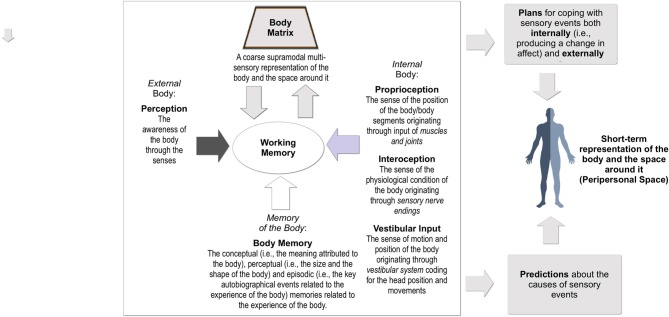
Multisensory integration of bodily signals.

The feeling of being a “real me” that emerges from one’s body—“bodily self-consciousness”—is one of the most complex experiences produced by the human mind (Moseley et al., 2012). In fact, it is the result of the multisensory integration (Figure 1) of many different sources of information (Riva, 2017b). The final outcome of this process, resulting from the connections between the posterior parietal cortex and the insular cortex, is a coarse supramodal multi-sensory representation of the body and the space around it, the “body matrix” (Moseley et al., 2012).

As recently noted by Barrett (2017): “For a brain to effectively regulate its body in the world, it runs an internal model of that body in the world” (p. 5). And this is the main goal of the body matrix: to predict (i) upcoming sensory events both inside and outside the body; (ii) how to use the body for dealing with them (Barrett, 2017). Specifically, its evolutive goal is to ensure resources to promote survival and reproduction (allostasis) by protecting and extending its boundaries at both the homeostatic and psychological level (Sterling, 2012; Barrett et al., 2016).

To produce the body matrix, data arriving from real-time multiple sensory modalities (exteroception; i.e., touch and vision) are first integrated with internal information (i.e., interoception and proprioception). The outcome of this process is then recalibrated according to predictions made using the stored information about the body from conceptual (i.e., the meaning attributed to the body), perceptual (i.e., the size and the shape of the body), and episodic (i.e., the key autobiographical events related to the experience of the body) memory.

The process of multisensory integration also imposes coherence on divergent bodily information by minimizing the amount of prediction errors (or “surprise”) about the expected sensory input (Hohwy, 2013). Specifically, the contents of the body matrix are adjusted evaluating the (dis)agreement (Talsma, 2015) between the perceived sensory activity, and the activity predicted through the integration of contents from different bodily and cognitive representations.

Once these errors are minimized, the body matrix provides predictions about the causes of sensory events and plans for coping with them both internally (i.e., producing a change in affect; Seth et al., 2012) and externally (i.e., by defining an action plan; Wang et al., 2014).

Every bodily sensation can have many different causes and effects. To recognize and cope with them in advance, our brain uses a complex process of predictive multisensory integration (Riva, 2017b).

In this view, the experience of the body is the result of a probabilistic process and, as such, may not reflect the characteristics of the physical body (Badoud and Tsakiris, 2017; Ferri et al., 2017). More, this vision suggests that the above multisensory processes do not influence the experience of the body only, but can also affect emotion regulation (Riva, 2017b; Tseng et al., 2015).

Specifically, emotions are not seen any more as iterative stimulus-response sequences, but as bodily simulations that, according to the individual’s previous experience, are labeled as emotions. As clarified by Barrett (2017): “That is, the brain constructs meaning by correctly anticipating (predicting and adjusting to) incoming sensations. Sensations are categorized so that they are: (i) actionable in a situated way; and therefore (ii) meaningful, based on past experience. When past experiences of emotion (e.g., happiness) are used to categorize the predicted sensory array and guide action, then one experiences or perceives that emotion (happiness).” (p. 9).

Finally, if both emotions and the experience of the body are the outcome of a complex process of predictive multisensory integration, their content will be impaired by a deficit in the process. In this view, damage, malfunctioning, or altered feedback from and toward the body matrix might be involved in the etiology of EDs. In the next pages we will try to deepen this point.

## Multisensory Impairments in Eating Disorders

As reported by various authors, individuals with EDs have emotional (Bydlowski et al., 2005; Lavender et al., 2015), visual (Engel and Keizer, 2017; Feusner et al., 2017), tactile (Keizer et al., 2011; Gaudio et al., 2014), proprioceptive (Case et al., 2012; Guardia et al., 2012) and interoceptive deficits (Badoud and Tsakiris, 2017; Klabunde et al., 2017).

Typically, these deficits have been assessed and examined specifically. However, as we have just seen, these different sources are all shaped by the process of predictive multisensory integration. More, we know that different processes (i.e., working memory), neurotransmitters (i.e., serotonin) and brain areas related to multisensory integration (i.e., frontal and parietal lobes) are impaired in individuals with EDs (Gaudio et al., 2016; Riva, 2016; Brooks et al., 2017).

This led us to hypothesize that these deficits may reflect a broader impairment in multisensory body integration (Zopf et al., 2016; Riva, 2017b; Riva and Gaudio, 2017). Specifically, following the emerging literature on this topic, it is possible to identify at least two different multisensory deficits (Riva, 2017b):

1. *An impairment in the ability to correctly link internal (interoceptive) bodily signals to their potential pleasant (or aversive) consequences*. According to Paulus and Stein (2010), a brain circuit that involves the medial prefrontal cortex, the dorsolateral prefrontal cortex and the anterior cingulate, evaluates anticipatory interoceptive signals using self-relevant and belief-based processes to identify those that are relevant. If this process is impaired, the individual can no longer correctly identify the relevant interoceptive signals that predict potential pleasant (or aversive) consequences. This situation produces significant impairments that affect the emotional abilities of the individual. First, it causes deficits in emotional clarity (i.e., recognizing and distinguishing between emotional signals) and emotional awareness (i.e., attention to emotions). Second, it impairs emotion regulation. Indeed, on one side, the individuals are not able to identify and use appropriate strategies to modulate the duration and/or intensity of emotional responses. On the other side, they experience greater difficulties in behavioral control when distressed and may decide to avoid emotion eliciting situations (Caslini et al., 2016). As noted by a recent review by Lavender et al. (2015), all these dimensions of emotion dysregulation can be found in both anorexia and bulimia nervosa.
2. *An impairment in the ability to update body memory (allocentric, offline) with new content from real-time perception-driven inputs (egocentric, online)*. As we have just seen, self-objectification is a critical risk factor for the development of EDs. But what is self-objectification from a neuropsychological view point? As we suggested previously (Riva et al., 2015) individuals objectify themselves when they use an allocentric frame of reference (i.e., using an observer’s viewpoint) to remember events in which they evaluate themselves based upon bodily appearance (i.e., my boyfriend telling me “You are fat. Your belly is so big”). To be included in the body matrix, these higher level long-term allocentric bodily memories have to be integrated with lower level real-time egocentric (i.e., first-person viewpoint) sensory signals (Mou et al., 2004). According to a prominent neural model of spatial memory and imagery (Byrne et al., 2007; Byrne and Becker, 2008), the translation between these representations involves the retrosplenial cortex, with the support of place and grid cells. In their own words: “Both encoding and retrieval/imagery require translation between egocentric and allocentric representations, which are mediated by posterior parietal and retrosplenial areas and the use of head direction representations in Papez’s circuit” (p. 340). If this process is impaired, the individual is not able to update the stored representation of the body with new real-time sensory data (Allocentric Lock). This situation produces significant impairments that affect the body experience of the individual (Serino et al., 2015). The first and most important is that the individual is no more able to modify/correct the autobiographical allocentric (observer view) memories of body related events (self-objectified memories) even if the body has changed (i.e., the memory of a fat belly, even if the belly is no more fat). This situation locks the individual to a permanent body shame that cannot be countered, pushing her/him towards EDs (Riva, 2012, 2014; Riva and Gaudio, 2012). More, these memories, through the predictive coding, structure the interpretation and acquisition of new ones (Riva et al., 2015). In simpler words, they generate a priming effect on any body related experience filtering from new experiences only the contents relevant to its meaning (i.e., the girl looking at my belly is smiling because it is fat).

## Restoring The Normal Functioning of Multisensory Body Integration

As we have seen before, the body matrix uses predictive coding and crossmodal processing, (i.e., the integration in a single representation of distinct inputs from sensorial modalities) to obtain coherence from discordant sensory information. This process suggests the possibility of updating the contents of the body matrix through the generation of new crossmodal associations between bodily stimuli that have not been previously experienced as systematically related. Specifically, crossmodal associations can produce crossmodal illusions (Bolognini et al., 2015): the contents of one sensory modality affect what we experience in another modality. In this context, crossmodal illusions may have two different roles. On one side, they can be used as assessment tools to verify an abnormal multisensory body integration. On the other side, they can reactivate and correct the contents of the body matrix. In fact, if the new crossmodal association is able to produce a significant prediction error (high surprise), it should be able to update the predictive internal models of the body matrix (Riva et al., 2017a).

It is interesting to note that “The body project”, the most effective and validated prevention program for EDs (Stice et al., 2011; Stice, 2016; Becker and Stice, 2017) developed by Eric Stice and his team, does not focus on eating or nutrition. Instead it uses a new affective/cognitive crossmodal association (negative emotions and the thin-ideal body standard to reduce thin-ideal internalization and appearance-related social comparison tendencies. Specifically in the program individuals voluntarily critique the thin-ideal standard of female beauty via verbal, written and behavioral exercises.

The appearance of new technologies and in particular the broader availability of virtual reality—a technology that has been already used successfully both to study body schema representations and distortions in normal and clinical population (Riva, 1998; Ferrer-García and Gutiérrez-Maldonado, 2010; Preston and Ehrsson, 2014, 2016; Mölbert et al., 2017), and in the treatment of EDs (Gutiérrez-Maldonado et al., 2016, 2017; Riva, 2017a)—offers new ways to provide crossmodal associations (Riva et al., 2017b; Tajadura-Jiménez et al., 2017; Weser et al., 2017) that can be used in clinical practice (Dakanalis et al., 2017b; Serino and Dakanalis, 2017). For example, different recent studies using virtual reality (VR)-based body swapping—a novel technology-based crossmodal association between vision (a new virtual reality [VR] body) and touch (the real body)—provided preliminary support to this approach, allowing a significant improvement in the ability to correctly estimate body size in both nonclinical (Preston and Ehrsson, 2014; Ferrer-Garcia et al., 2017) and clinical samples (Keizer et al., 2016; Serino et al., 2016a,b).

At the moment these bodily illusions provide a short-term effect only (Keizer et al., 2016). However, this result may be improved by using more advanced crossmodal associations that also target inner body signals (i.e., interoceptive, proprioceptive and vestibular signals). To reach this goal, VR has to be integrated with bio/neuro-feedback and/or brain/body stimulation technologies able to measure and modulate the internal body experience (Riva et al., 2017a). For example, the use of heart rate variability and respiratory sinus arrhythmia biofeedback can be used to enhance interoceptive sensitivity and the appraisal of interoceptive bodily signals (Herbert et al., 2013; Tajadura-Jiménez and Tsakiris, 2014).

## Discussion

Shape and weight overvaluation are considered core symptoms of EDs. In this article, we suggested that the transdiagnostic centrality of shape and weight overvaluation in individuals with EDs may reflect a broader impairment in multisensory body integration. Multisensory body integration is a critical cognitive and perceptual process, allowing the individual to protect and extend her/his boundaries at both the homeostatic and psychological levels (Riva, 2017b). More, it is not an innate ability but the result of a long developmental process, reaching maturity only at the age of 10–11, through which the individual is able to define and compare her/his own body in relation to an ideal cultural body defined by institutional norms and values (Riva, 2017b). As discussed before, this process integrates sensory data arriving from real-time multiple sensory modalities and internal information with predictions made using the stored information about the body from conceptual, perceptual and episodic memory.

In this vision, multisensory integration deficits represent a failure in this functional adaptation process, which may impair the emotional and bodily experience of the individual.

Starting from the above premises, in this article we provided a plausible and testable theory of the etiology of EDs based on impairments in:

1. The ability to link internal (interoceptive) bodily signals to their potential pleasant (or aversive) consequences, and
2. The ability to update body memory (allocentric, offline) with new content from real-time perception-driven inputs (egocentric, online).

This theory has three strengths. First, it offers a plausible and testable model able to explain the etiology of these disorders. Specifically, to test it we need to assess multisensory integration deficits in EDs and use a longitudinal study to examine the influence of this vulnerability factor in predicting both the onset and maintenance of (DSM-5) EDs in the long term.

Second, it provides the rationale for a novel multisensory technology aiming at restoring the normal functioning of these processes. Specifically, it provides a rationale for using new technologies (i.e., virtual reality and brain/body stimulation) to target crossmodal associations to reactivate and correct multisensory body integration processes.

A final key strength of this vision is that it proposes a translational neuroscience perspective, effectively integrating some of the existing prominent etiological models of EDs. On one side, multisensory deficits produce a dysfunctional system for evaluating self-worth in line with the assumptions of the transdiagnostic cognitive behavioral theory (Fairburn, 2008; Cooper and Fairburn, 2011) models. Moreover, the critical role of stress and anxiety related issues in producing multisensory body integration deficits is in agreement with the revised cognitive-interpersonal maintenance model and the theory of emotional eating (Schmidt and Treasure, 2006; Treasure and Schmidt, 2013). Finally, it allows us to better explain the role of self-objectification in EDs: the onset and maintenance of EDs is not related to the experience of self-objectification in itself but to the impossibility of countering the body shame produced by it, even when the real body of the subject is in line with the thin-ideal standard of beauty.

In conclusion, although various studies support a possible role of multisensory body integration deficits in the etiology of EDs and link them to body and emotional problems in individuals with EDs there are still numerous open questions: What are their causes? Are they different for the individual EDs? Are there ways to protect against them? Are there gender differences? How can we modify them? Extensive new interdisciplinary research is required to confirm this hypothesis and to provide meaningful answers to all these questions.

## Author Contributions

GR conceived and developed the initial draft, contributed to the enhancement of the original draft, and participated in developing the final draft; AD worked with GR to enhance the original draft and develop it into the final draft; both authors have reviewed and approved the final manuscript as submitted.

## Conflict of Interest Statement

The authors declare that the research was conducted in the absence of any commercial or financial relationships that could be construed as a potential conflict of interest.
